# Nematicidal Activities of Saccharin and Erythritol Against Pinewood Nematode

**DOI:** 10.2478/jofnem-2022-0038

**Published:** 2022-09-30

**Authors:** Junheon Kim, Sujin Lee

**Affiliations:** 1 Forest Entomology and Pathology Division, National Institute of Forest Science, Seoul 02455, Republic of Korea

**Keywords:** *Bursaphelenchus xylophilus*, management, pine wilt disease, *Pinus*, sweetener

## Abstract

The pinewood nematode (PWN), *Bursaphelenchus xylophilus* (Steiner & Bührer), causes pine wilt disease (PWD) resulting in severe environmental damage to pine forest ecosystems worldwide. To develop alternative strategies for managing PWD, the nematicidal activities of two sweeteners, erythritol and saccharin, were investigated. Among these two sweeteners, saccharin induced higher mortality in a dose-dependent manner. The LC_50_ and LC_90_ values of saccharin were estimated to be 0.321 M and 0.615 M, respectively. However, erythritol did not exhibit nematicidal activities. The results of our study demonstrated that saccharin is lethal to PWN and shows nematicidal effects in a dose-dependent manner. Although the mechanisms of saccharin toxicity are not yet investigated, saccharin could be used as an effective alternative for the management of PWN.

The pinewood nematode (PWN), *Bursaphelenchus xylophilus* (Steiner & Bührer), causes pine wilt disease (PWD), which results in severe environmental damage to pine forest ecosystems over multiple continents, including Asia and Europe (Mota and Vieira, 2008; Zhao et al., 2008; Zamora et al., 2015). As *Pinus* species are the predominant tree species in Korean forests and are highly susceptible to PWN, ecological and economic damage are considerably high.

The current strategy for the management of PWD in Korea is avermectin-class nematicide injections in the pine trunk for PWD prevention (Korea Forest Service, 2017). Although some advantages of avermectin-class nematicide injection are known, improvements in nematicide injection are required owing to various associated disadvantages, such as high cost and possible development of nematicide resistance. Therefore, the nematicidal activities of several alternatives, such as the use of extracts of microorganisms, essential oils of plants, and plant extracts, have been investigated (Liu et al., 2016; Guo et al., 2017; Cha et al., 2019; Kang et al., 2022).

Recently, the insecticidal activity of non-nutritive sweeteners, such as erythritol and saccharin, has been reported (O’Donnell et al., 2016; Zheng et al., 2016; Choi et al., 2017; Fisher et al., 2017; Zhang et al., 2017; Wentz et al., 2020; Schmidt-Jeffris et al., 2021). Since sweeteners are highly water-soluble and PWN inhabits water circumstance in a tree, the sweeteners are expected to affect the survival of PWN.

In this study, we examined the dose-dependent effects of two sweeteners, erythritol and saccharin, on the survival of PWN.

## Materials and Methods

### Nematodes

*Bursaphelenchus xylophilus* was provided by the National Institute of Forest Science, Republic of Korea, and identified by morphological characteristics and genetic differences using the restriction fragment length polymorphism (RFLP) method (Han et al., 2008). The nematodes were reared on a fungal mat of *Botrytis cinerea* established on potato dextrose agar medium at 25 ± 1°C and 40% relative humidity (RH) for several generations.

### Nematicidal activities of two sweeteners on *B. xylophilus*

**Figure 1:**
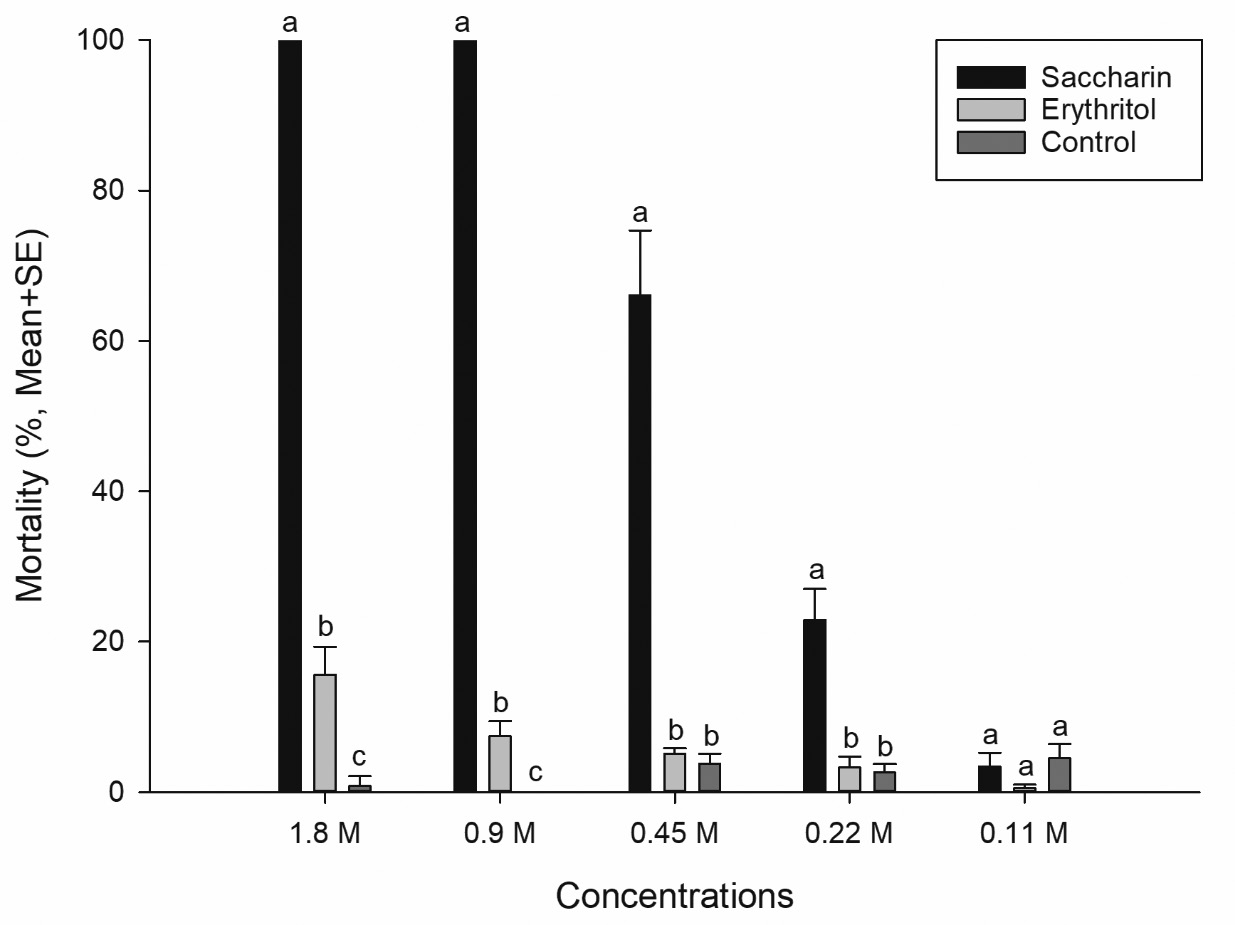
The nematicidal activity of saccharin and erythritol against *Bursaphelenchus xylophilus*. Data represent the mean + SE of mortality at each concentration. The same letters within in a concentration are not significantly different (Tukey’s honest significant difference test at *P* = 0.05, *n* = 10, except 0.11-M concentration, which was *n* = 5).

Two sweeteners, saccharin (molecular weight: 183.18, purity: ≥99.9%; Hanseung Food Co. Ltd., Gimpo, Korea) and erythritol (molecular weight: 122.12, purity: ≥99%; Sigma-Aldrich, St. Louis, MO) (Fig. 1), were dissolved in distilled water to prepare a solution having a 2-M concentration. The 2-M solution was serially diluted to obtain solutions having final concentrations of 1.8 M, 0.9 M, 0.45 M, 0.23 M, and 0.11 M. Each treatment set comprised four replicates, and each treatment set was repeated 10 times except the 0.11-M solution. The test set with a 0.11-M solution was repeated five times. Nematodes treated with distilled water were used as the control set. Working solutions were prepared and used on the day of the experiments. Approximately 1,000 nematodes in 90 mL of distilled water were placed in each well of a 96-well cell culture plate, and then 10 mL of the test solution was added. For the 1.8-M test solution, ca. 1,000 nematodes in 10 mL of distilled water were added to 90 mL of 2-M solution of the sweetener. Then, the 96-well cell culture plates were maintained at 25 ± 1°C and 40% RH in the dark, and nematode mortality was determined at 24 hr after treatment. After thorough mixing of the solution in the wells using a micropipette, 10 mL of the treatment solution was taken and transferred to 10 mL of clean water on a slide glass for counting nematodes. After repeating the last step twice, the total of dead nematodes was recorded to calculate mortality. Nematodes were considered dead if their bodies were straight and motionless, even after being transferred to clean water. Nematicidal activity was classified according to mortality as follows: strong (80%–100% mortality), moderate (60%–80% mortality), weak (40%–60% mortality), and no activity (<40% mortality).

### Statistical Analysis

Mortality data were corrected using Abbott’s formula (Abbott, 1925), and the corrected mortality was arcsine square root-transformed for one-way analysis of variance (ANOVA). The means were compared and separated using the Tukey–Kramer HSD test. The lethal dose (LD) values were estimated by probit analysis using dose-response data. Statistical analyses were performed using JMP ver. 9.0.2 (SAS Institute Inc., Cary, NC). The mean (± SE) values of the untransformed data were recorded. The untransformed data are reported.

## Results and Discussion

The mortality of *B. xylophilus* was significantly affected by the presence of saccharin at 1.8-M concentration (one-way ANOVA, *F*_2,27_ = 313.30, *P* < 0.0001; Fig. 1). The mortality rate of saccharin was dose-dependent. Saccharin solution showed strong nematicidal activity at 0.9-M and 1.8-M concentrations, and moderate activity at 0.45-M concentration. However, below 0.23 M concentration, it showed no activity. Based on the dose-response data, a lethal concentration (LC) value was obtained. The LC_50_ and LC_90_ values of saccharin were 0.321 M (95% confidence limit [CL]: 0.309–0.333) and 0.615 M (95% CL: 0.583–0.654), respectively (slope ± SE: 1.97 ± 0.07, c^2^ = 580.17, df = 48).

In our study, erythritol did not exhibit nematicidal activity. However, erythritol showed a higher mortality rate than other sweeteners, such as sucrose, against dipteran insect pests (Zheng et al., 2016; Choi et al., 2017), psylla (Wentz et al., 2020), ant (Zhang et al., 2017), and mite (Schmidt-Jeffris et al., 2021). As erythritol is a non-nutritive sugar, it cannot be used as a substrate for enzymes involved in sugar metabolism. Choi et al. (2017) suggested that mortality by erythritol would result from starvation due to intake of non-metabolizable erythritol or experiencing abnormally high osmotic pressure in the hemolymph with erythritol diffused from the midgut. Fisher et al. (2017) suggested that the decreased survival rates induced by erythritol could be the result of starvation rather than insecticidal activity. Erythritol in the tissues is not always toxic to arthropods. Some insect species that are seasonally exposed to freezing conditions produce erythritol and other polyhydric alcohols as tissue cryoprotectants (Danks, 2004; Kostal et al., 2007). Therefore, the differential erythritol-induced mortality reported in insect pests and PWN may result from the difference in utilization of sugar as food or the environmental conditions of PWN that overwinter under freezing conditions.

**Figure 2:**
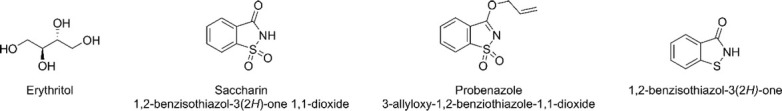
Structure of erythritol, saccharin, probenazole, and 1,2-benzisothiazol-3(2*H*)-one.

Unlike erythritol, saccharin induced higher mortality rates in our experiments. Although mortality by saccharin has been studied less than that caused by erythritol, previous studies have shown results similar to those of our study, i.e. results demonstrating that saccharin affected the survival of PWN. Zheng et al. (2016) reported higher mortality induced by saccharin than those by sucrose, fructose, and glucose, but less than the mortality induced by erythritol, against *Bactrocera dorsalis* (Diptera: Tephritidae). Moreover, saccharin induced behavioral changes such as a reduction in the frequency of flights and walks. Similar to erythritol, saccharin affected the survival of *Solenopsis invicta* (Hymenoptera: Formicidae) and induced relatively high mortality (Zhang et al., 2017). Sharma et al. (2020) reported that saccharin did not affect the survival, lifespan, fecundity, or egg hatching of *Aedes aegypti* (Diptera: Culicidae). Saccharin, 1,2-benzisothiazole-3(2*H*)-one 1,1-dioxide (BHT), is a metabolite of probenazole (Fig. 2), an effective fungicide (Uchida et al., 1973). Although the direct fungicidal activity of saccharin has not been reported, it is known to induce resistance to fungi in some plants (Nakashita et al., 2002; Phuong et al., 2020; Mejri et al., 2021). 1,2-Benzisothiazoline-3-one (Fig. 1), an analog of saccharin, showed remarkable inhibitory effects on the growth of microorganisms such as bacteria, fungi, and algae (Silva et al., 2020).

To conclude, our study has unequivocally demonstrated that saccharin is lethal to PWN and that the nematicidal effects of saccharin are dose-dependent. Although the nematicidal mechanism of saccharin has not yet been investigated, it could be used as an alternative for the management of PWN. Further studies are needed to examine in vivo effects of saccharin on PWN in living pine trees.
